# lncExplore: a database of pan-cancer analysis and systematic functional annotation for lncRNAs from RNA-sequencing data

**DOI:** 10.1093/database/baab053

**Published:** 2021-08-31

**Authors:** Yi-Wei Lee, Ming Chen, I-Fang Chung, Ting-Yu Chang

**Affiliations:** Institute of Biomedical Informatics, National Yang Ming Chiao Tung University, No.155, Sec. 2, Linong St., Beitou District, Taipei 11221, Taiwan; Department of Genomic Medicine and Center for Medical Genetics, Changhua Christian Hospital, No.176, Chong-Hua Rd., Changhua 50046, Taiwan; Research Department, Changhua Christian Hospital, No.135, Nan-Hsiao St., Changhua 50006, Taiwan; Department of Genomic Science and Technology, Changhua Christian Hospital Healthcare System, No.176, Chong-Hua Rd., Changhua 50046, Taiwan; Department of Obstetrics and Gynecology, Changhua Christian Hospital, No.135, Nan-Hsiao St., Changhua 50006, Taiwan; Department of Medical Genetics, National Taiwan University Hospital, No.7, Chung Shan S. Rd.(Zhongshan S. Rd.), Zhongzheng Dist., Taipei 10041, Taiwan; Department of Obstetrics and Gynecology, College of Medicine, National Taiwan University, No.7, Chung Shan S. Rd.(Zhongshan S. Rd.), Zhongzheng Dist., Taipei 10041, Taiwan; Department of Biomedical Science, Dayeh University, No.168, University Rd., Dacun, Changhua 51591, Taiwan; Department of Medical Science, National Tsing Hua University, No.101, Section 2, Kuang-Fu Road, Hsinchu 30013, Taiwan; Institute of Biomedical Informatics, National Yang Ming Chiao Tung University, No.155, Sec. 2, Linong St., Beitou District, Taipei 11221, Taiwan; Center for Systems and Synthetic Biology, National Yang Ming Chiao Tung University, No.155, Sec. 2, Linong St., Beitou District, Taipei 11221, Taiwan; Department of Genomic Medicine and Center for Medical Genetics, Changhua Christian Hospital, No.176, Chong-Hua Rd., Changhua 50046, Taiwan; Research Department, Changhua Christian Hospital, No.135, Nan-Hsiao St., Changhua 50006, Taiwan; Department of Genomic Science and Technology, Changhua Christian Hospital Healthcare System, No.176, Chong-Hua Rd., Changhua 50046, Taiwan; Department of Bioscience Technology, Chung Yuan Christian University, No.200, Chung Pei Road, Chung Li District, Taoyuan 32023, Taiwan

## Abstract

Over the past few years, with the rapid growth of deep-sequencing technology and the development of computational prediction algorithms, a large number of long non-coding RNAs (lncRNAs) have been identified in various types of human cancers. Therefore, it has become critical to determine how to properly annotate the potential function of lncRNAs from RNA-sequencing (RNA-seq) data and arrange the robust information and analysis into a useful system readily accessible by biological and clinical researchers. In order to produce a collective interpretation of lncRNA functions, it is necessary to integrate different types of data regarding the important functional diversity and regulatory role of these lncRNAs. In this study, we utilized transcriptomic sequencing data to systematically observe and identify lncRNAs and their potential functions from 5034 The Cancer Genome Atlas RNA-seq datasets covering 24 cancers. Then, we constructed the ‘lncExplore’ database that was developed to comprehensively integrate various types of genomic annotation data for collective interpretation. The distinctive features in our lncExplore database include (i) novel lncRNAs verified by both coding potential and translation efficiency score, (ii) pan-cancer analysis for studying the significantly aberrant expression across 24 human cancers, (iii) genomic annotation of lncRNAs, such as *cis*-regulatory information and gene ontology, (iv) observation of the regulatory roles as enhancer RNAs and competing endogenous RNAs and (v) the findings of the potential lncRNA biomarkers for the user-interested cancers by integrating clinical information and disease specificity score. The lncExplore database is to our knowledge the first public lncRNA annotation database providing cancer-specific lncRNA expression profiles for not only known but also novel lncRNAs, enhancer RNAs annotation and clinical analysis based on pan-cancer analysis. lncExplore provides a more complete pathway to highly efficient, novel and more comprehensive translation of laboratory discoveries into the clinical context and will assist in reinterpreting the biological regulatory function of lncRNAs in cancer research.

**Database URL**: http://lncexplore.bmi.nycu.edu.tw

## Introduction

Over the past few decades, cancer research has mainly focused on identifying the protein-coding genes that are causally linked to the tumorigenesis and deregulation of biological processes in human cells. However, the development of next-generation sequencing (NGS) technologies has provided researchers more opportunities to clarify the complex transcriptional landscape in human cells and to focus on another type of important functional RNAs, namely non-coding RNAs (ncRNAs). Although ncRNAs do not encode proteins, ncRNAs have been shown to participate in a diverse collection of regulatory pathways in mammals (1). Among various types of ncRNAs, such as microRNAs (miRNAs), transfer RNAs (tRNAs), ribosomal RNAs (rRNAs), and long non-coding RNAs (lncRNAs), lncRNAs represent a prevailing and functionally diverse class of the transcriptome with a sequence size ranging from 200 bases to approximately 100 000 bases (2). In contrast to protein-coding transcripts, lncRNAs constitute a large and diverse class of non-coding transcripts, which have low sequence conservation across species (3, 4) and express at lower levels (5). In addition, lncRNAs have high tissue specificity and have been implicated in chromatin modification, transcriptional gene regulation and post-transcriptional gene regulation through functioning as important positive and negative modulators of protein-coding gene expression (3, 6–8). Furthermore, many studies recently demonstrated that some lncRNAs regulate the neighbor target coding genes in *cis* (9–12). This implies that lncRNAs may possess diverse functions in regulating human biological processes (2).

Although previous studies once considered lncRNAs to be transcriptional noise or cloning artifacts, the recent accumulating findings have suggested that lncRNAs act as key regulators in many crucial biological processes including X-inactivation, cell differentiation, RNA processing and modification, DNA methylation and others (13–16). Moreover, lncRNAs are emerging as new players as one of the critical regulatory components in the transcription process, such as molecular signals, decoys, guides and scaffolds (2, 17, 18). Recently, increasing evidence has shown that dysfunction and dysregulation of lncRNAs are associated with various human diseases, especially cancers, and further shown that mechanisms of lncRNAs contribute to the complex etiology of disease (19–21). The relationship between lncRNAs and cancer has been revealed in some case studies. Some of the well-characterized lncRNAs reveal a functional role in gene expression regulation that may occur in *cis*-gene (genomically local) or in *trans*-gene (genomically distant) contexts. In breast cancer, the presence of lncRNA *BCAR4* causes estrogen-independent growth and antiestrogen resistance (22). Furthermore, the expression level of lncRNA *BCAR4* is associated with poor metastasis-free survival (MFS) (23). The abnormally upregulated well-known imprinted lncRNA *H19* contributes to cell proliferation in breast cancer and bladder cancer (24, 25). Additionally, some lncRNAs could be classified as enhancer RNAs (eRNAs) which are transcribed unidirectionally from enhancer regions in general (26). eRNAs have been shown to play functional roles in chromatin interactions or be part of super-enhancers at target promoter regions and also to be correlated with expressions of nearby genes (27). Thus, the dysregulated expression of lncRNAs in cancer marks the trajectory of disease progression and serves as potential candidate biomarkers for patient outcomes.

In this context, as the importance of lncRNA regulation of disease progression has been reported, an increasing number of studies have devoted great effort to identifying lncRNA functions. For instance, there are over 9640 human genome loci classified as lncRNAs since GENCODE project v7 (5). However, a great number of lncRNAs must be further characterized in depth to determine their regulatory roles in cells. Thus, several well-known lncRNA databases, such as NONCODE v5 and NRED, collect and classify basic information on lncRNAs (28, 29). The database NONCODE v5 contains 548 640 transcripts from 17 species and provides a comprehensive repository for varying aspects of lncRNA annotations, such as expression profiles based on the human body map project and literature-based disease–lncRNA associations (29). NRED provides lncRNA expression information in humans and mice from microarray and *in situ* hybridization data (28). In addition, several annotation databases provide information regarding lncRNA association with diseases. These databases include Lnc2Cancer, lncRNAdb, lncRNAtor, lncRNADisease and EVlncRNAs (30–34). Some databases have also been developed to categorize additional molecular associations. For example, DIANA-lncBase and starBase provide miRNA recognition elements for human lncRNAs by combining the Crosslinking and immunoprecipitation sequencing (CLIP-seq) validations and *in silico* target predictions (35, 36). All the databases mentioned above provide comprehensive lncRNA information, such as expression profile, published experimental validated disease association and molecular relationships. These databases were well-designed for their respective specific purpose [disease-focused (lncRNA2Cancer, lncRNADisease and EVlncRNAs), miRNA-interaction-focused (DIANA-lncBase and starBase) and expression-focused (NONCODE v5)] and are mostly limited to the known lncRNAs. However, there are a vast number of lncRNAs whose exact functions are unknown. Therefore, researchers require an integrative database to gain deeper understanding of the molecular mechanisms of lncRNAs. Thus, a comprehensive resource and integrative interface to explore lncRNA annotations and clinical information is still lacking. In addition, high-throughput sequencing technology and computational methodologies have provided opportunities to predict novel lncRNAs and promote understanding of the regulatory functions of lncRNAs in human cancers. Therefore, with the amount of available public RNA-sequencing (RNA-seq) data, it has become urgently important for researchers seeking to understand the diverse biological functions of lncRNAs and identifying the potential cancer-related biomarkers to have streamlined access to these comprehensive data existing in numerous disparate databases in order to be able to systematically analyze and cross-reference lncRNAs in detail with expression profiles, novel transcripts, disease specificity and clinical information. Our lncExplore database is intended to fulfill this urgent need.

Large-scale genomics projects, such as The Cancer Genome Atlas (TCGA) and GENCODE, give us opportunities to make available potentially unconventional biomarkers through systematically organizing the massive public genomic data (i.e. RNA-seq data) and clinical information (5, 37). In this context, to fulfill the need for better understanding the molecular mechanisms of lncRNAs and assessing the exact functions of lncRNAs, we have built the user-friendly lncExplore database to help users understand the regulatory role of lncRNAs in cancers and identify potential cancer-specific lncRNA biomarkers. Understanding how lncRNAs contribute to cancer-specific biomarkers or regulatory roles has thus become a remarkable area of study (38). In our lncExplore database, we not only collect the known lncRNAs downloaded from Ensembl and GENCODE but also first collect the unique novel lncRNAs that have been predicted from a large amount of RNA-seq data. In order to get reliable predicted lncRNAs, those novel lncRNAs were further validated by their corresponding coding potential score and translation efficiency score. Accordingly, these novel lncRNAs were annotated based on the coding potential score, the corresponding expression profile and the disease specificity across 24 cancers. Therefore, lncExplore supports pan-cancer analysis aimed at observing the potential lncRNA–cancer associations. Gene Ontology (GO) enrichment analysis was performed to characterize the associated molecular role of unannotated lncRNAs. We further utilized an *in silico* approach to identify eRNAs by overlapping the lncRNAs with the enhancer regions, which provide a valuable comparative module in dissecting the functional link between eRNAs and nearby genes (39, 40). lncExplore also provides a user-friendly interface to allow users to discover potential lncRNA biomarkers across different cancers as well as cancer-specific analysis and further provides the related biological regulatory information about lncRNAs. Ultimately, with the comprehensive molecular annotations and valuable clinical information of known and novel lncRNAs, we believe that lncExplore integrates and summarizes multiple meaningful correlations between molecular research and clinical disciplines.

## Results

### Database overview

**Figure 1.**
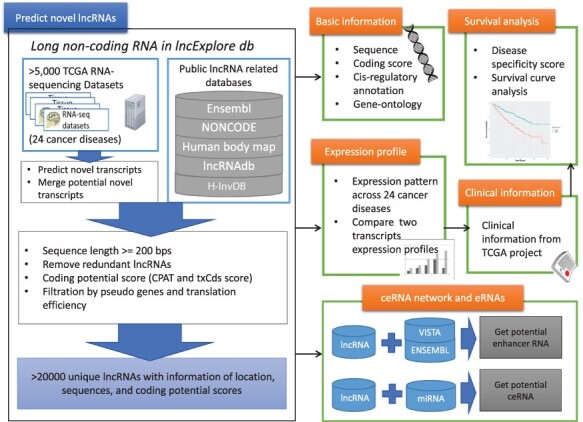
Schematic representation of lncExplore database. The available known lncRNA resources were collected from five public databases (Ensembl, Human body map, NONCODE, H-InvDB and lncRNAdb). We predicted the novel transcripts from RNA-seq datasets. To eliminated false-positive novel lncRNAs, we only collected the transcripts with following characteristics: sequences longer than 200 nucleotides, sequences with low coding potential probability, sequences without potential pseudogenes and sequences with similar translation efficiency as known lncRNAs. The elimination step reduced transcripts into >20 000 ‘unique lncRNA transcripts’ in our database. In lncExplore, the information about lncRNAs includes basic genomic information, gene expression profiles across cancers, predicted molecular annotations (GO, eRNA and ceRNA) and clinical-related information (disease specificity score and survival curve).

We designed a user-friendly query interface for lncExplore to provide statistical visualization and annotation detail to present the results from pan-cancer analysis based on RNA-seq and ribosome-profiling sequencing (Ribo-seq) data for novel lncRNAs. The lncExplore database is composed of four main modules of data representation (Figure 1): (i) basic genomic information and enrichment analysis results, such as sequence, *cis*-regulatory role and gene ontology, for known and novel lncRNAs; (ii) lncRNA expression profiles across 24 human cancers based on TCGA RNA-seq data; (iii) lncRNA-related clinical annotation such as cancer specificity and survival curve analysis; and (iv) observation of the regulatory roles as enhancer RNAs (eRNAs) and competing endogenous RNAs (ceRNAs). Our database not only collects known lncRNAs from Ensembl and GENCODE but also records 22 428 potential unique novel lncRNAs that have been predicted from 5034 RNA-seq datasets and then identified by coding potential and translation efficiency scores. As a result, lncExplore consists of a web-based integrated platform bridging the gap between basic lncRNA statistical information from pan-cancer analysis and the corresponding clinical information regarding different human cancers.

### Database content

#### Novel and known lncRNA populations

**Figure 2.**
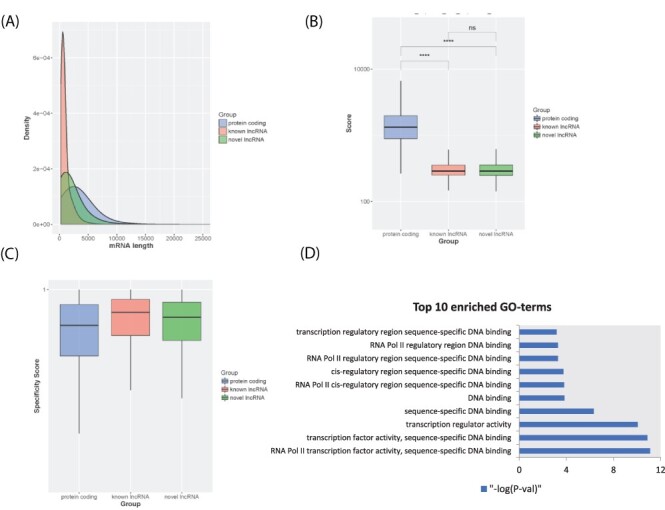
LncRNA transcript characterization. (A) Distribution of transcript lengths for known lncRNAs (red), novel lncRNAs (green) and protein-coding genes (blue). (B) Boxplot of coding potential of transcripts by txCDS tool computed in three sets: known lncRNAs (red), novel lncRNAs (green) and protein-coding genes (blue). The statistical significance difference was calculated using Wilcoxon rank sum test. ‘ns’: non-significant, ‘****’: *P*-value < 0.001. (C) Boxplot of disease specificity score in three sets: known lncRNAs (red), novel lncRNAs (green) and protein-coding genes (blue). (D) Top 10 statistically significant enriched GO terms (molecular function category) for lncRNA *HOTTIP* 12 adjacent genes.

In lncExplore, we collected 23 898 known lncRNAs from Ensembl and GENCODE databases and additionally predicted potential novel lncRNAs from TCGA RNA-seq data. The sizes of known lncRNAs in lncExplore range from 200 to 91 667 bp with an average of 967 bp. Moreover, in order to retrieve the unique novel lncRNAs and filter out the redundant data, we compared those lncRNA sequences with five well-known lncRNA databases including Ensembl, NONCODE v5, lncRNAs db, H-InvDB v9 and human body map project (29, 41–43). To systematically identify the potential lncRNAs from novel transcripts predicted from RNA-seq data in our novel lncRNA identifying pipeline, we integrated a variety of features including coding potential and ribosome occupancy estimation of transcripts to discard protein-coding transcripts. There are 511 574 unique novel lncRNA candidates with low coding potential scores. Furthermore, we consolidated Ribo-seq datasets, which contain information about actively translated mRNA, to filter out candidate lncRNAs with evidence of coding potential. A total of 25 249 novel lncRNAs were kept by our pipeline. In addition, in order to remove potential pseudo genes, novel lncRNA candidates, which have high sequence similarity compared with GENCODE protein-coding genes, were removed from our database. Finally, our lncExplore has collected a total of 22 428 novel unique lncRNAs with sizes ranging from 200 to 37 396 bp with an average of 1806 bp (Figure 2A). The average of coding potential score estimated by txCds is 327.78 and 312.86 for known lncRNAs and novel lncRNAs, respectively (Figure 2B). Moreover, the average of coding probability estimated by coding-potential assessment tool (CPAT) is 0.1032 and 0.0811 for known lncRNAs and novel lncRNAs, respectively. Furthermore, lncExplore provides the tissue specificity score for each unique novel lncRNA according to the tissue-specific property of lncRNAs (44). We also demonstrated that lncRNAs have higher tissue specificity scores than protein-coding genes by analyzing the expression profiles across cancer diseases in our database (Figure 2C). In addition, there are 6585 and 15 843 novel lncRNAs (29.4% and 70.6%) located in the intragenic and intergenic regions, respectively.

#### *Cis*-regulatory information and GO annotations of lncRNAs

In lncExplore, we infer the molecular annotation of human lncRNAs based on the *cis*-regulatory rules. A total of 19 638 known lncRNAs and 19 551 novel lncRNAs were identified for possibly regulating potential targeted protein-coding genes by screening 100 kb upstream/downstream region of lncRNAs (9, 45). We performed the GO enrichment analysis in all categories of GO terms (including biological process: BP, cellular component: CC and molecular function: MF) with the *cis*-lncRNAs’ neighbor-coding genes. The results of GO enrichment analysis were provided to illustrate the potential regulatory functions of lncRNAs. For example, through searching neighbor-coding genes and GO enrichment analysis, we found that there are >20 statistically significant (‘Fisher’s’ exact test, *P*-value < 0.05) GO terms in the lncRNA *HOTTIP* neighbor-coding genes, including RNA polymerase II binding, transcription activity, DNA binding (Figure 2D), etc. Some evidence for those related regulatory functions of lncRNA *HOTTIP* has been shown as follows. lncRNA *HOTTIP* is brought into close proximity of its targeted *HOXA* gene by chromosomal looping (11). lncRNA *HOTTIP* binds to WDR5 protein, a component of WDR5/MLL histone methyltransferase complex. This complex regulates the expression of *HOXA* genes by activating trimethylation of histone H3 at lysine 4 (H3K4me3) marks of *HOXA* genes. Additionally, *HOTTIP* plays an important role in the promotion of cell proliferation by regulating the expression of its neighboring *HOXA* genes in various types of human cancers (46–48). Therefore, through *cis*-regulatory mechanisms with neighbor-coding genes, we can find potential biological functions for known/novel lncRNAs in lncExplore.

#### lncRNAs as eRNAs or ceRNAs

To investigate the potential regulatory roles for lncRNAs, we further predicted that some lncRNAs may play roles as eRNAs or ceRNAs. These eRNAs, which also act as *cis*-regulatory elements (such as the components of super-enhancers), actively engage in promoting nearby mRNA expression in transcription regulation (2, 49). A total of 7444 lncRNAs (3606 known and 3838 novel lncRNAs) were identified as eRNAs by comparing the genomic coordinates of lncRNAs overlapped with the known enhancer regions from Ensembl and VISTA enhancer databases. For example, lncRNA *CCDC26* was found to be a novel biomarker in acute myeloid leukemia and also found to control myeloid leukemia cell growth (50, 51). Using our *in silico* analysis method, we discerned that *CCDC26* is nearby (enhancer of the gene) the hs1709 (VISTA ID) enhancer region. The flanking region of hs1709 includes the protein-coding gene *GSDMC* that has an upregulated expression in some cancers. This information could provide users a link to identify the putative indirectly regulatory relationship between lncRNA *CCDC26* and the potential target gene *GSDMC*.

In addition to behaving as eRNAs, lncRNAs can serve as ceRNAs to regulate the targeted mRNA expression in post-transcriptional regulations. Here each novel lncRNA–miRNA association was predicted by TargetScan with default parameters and the complementary sites of targeted lncRNAs were listed as a table (52). In lncExplore, we recorded novel lncRNA–miRNA interactions from 2588 known miRNAs and 22 428 novel lncRNAs. For example, based on the TargetScan predicted results for the known lncRNA *UCA1*, there are 1994 potential *miRNA*–*UCA1* pairs in our database. Furthermore, *UCA1* can absorb miR-203 and indirectly influence the regulatory efficiency of *miR-203* for its targeted genes. Through the ceRNA network, *UCA1* can regulate the expression of miR-203-targeted transcript *ZEB2* that is a transcription factor related to tumor metastasis (53).

### Web interface

#### Novel lncRNAs predicted from RNA-seq data

**Figure 3.**
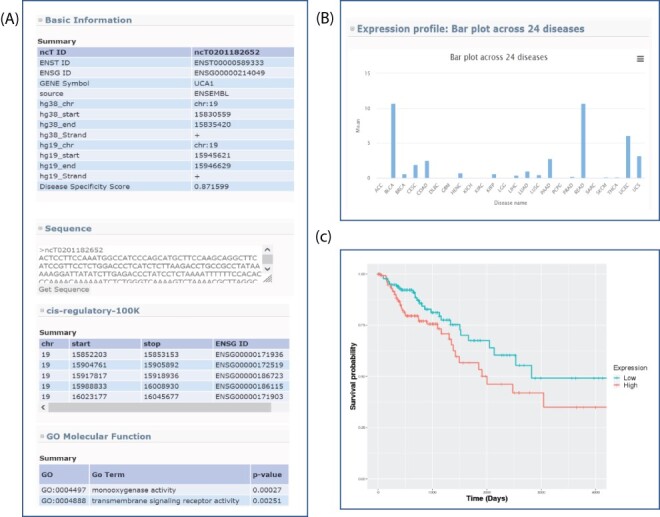
Sample output for searching lncRNA *UCA1*. (A) Basic genome information for each lncRNAs including genomic information, sequence, potential *c*is-regulatory elements and GO in the lncExplore database. (B) The average of expression values (FPKM) across 24 diseases were represented by bar charts. (C) Kaplan–Meier plot of lncRNA *UCA1* in colon adenocarcinoma was displayed in different colors for two groups (high and low expression), respectively.

All of the novel lncRNAs recorded in lncExplore can be queried based on a user-defined genome region or the adjacent region of known genes. In our result pages, a summarized table of lncRNAs was presented to reveal basic lncRNA information such as genome location, gene symbol, disease specificity score and lncRNA sequence (Figure 3A). Moreover, the disease specificity score will help users find the cancer-specific novel lncRNAs more easily.

#### Differential expression and statistical results for lncRNAs among 24 human cancers by pan-cancer analysis

lncExplore provides graphical visualization of lncRNA expression profiles across different cancers. This feature helps users examine expression patterns of the interested lncRNAs by providing gene symbol, Ensembl transcript ID, Ensembl gene ID or user-defined chromosome region. For example, by entering a specific transcript’s ID on the search page, users could obtain an overview of the summarized expression profiles of the selected lncRNA among 24 cancer types. All of these expression values estimated in fragments per kilobase of transcript per million mapped reads, (FPKM) were estimated from RNA-seq data. Moreover, the expression profiles are presented for different cancer types by bar plot (Figure 3B). These readily understood visualizations will assist users to easily compare the lncRNA expression pattern across different cancers. Furthermore, lncExplore also allows users to simultaneously enter the lncRNA ID and coding gene ID of interest. Users can identify the transcriptional regulatory roles of lncRNAs on silencing/activating the nearby genes by the comparison of two lncRNA expression patterns in each human cancer using scatter plot and Pearson correlation coefficient value. The comparison interface in our result pages is able to provide evidence for the lncRNA-related regulatory phenomenon in different cancers. For pairwise comparison (normal datasets vs. tumor datasets), users could select their interesting lncRNA among six diseases in our database. Then, the comparison result was represented by scatter plot.

#### Tissue specificity score for lncRNAs

In lncExplore, a ‘Tau’ score (44) was used to indicate whether the lncRNA signature is restricted to specific tissues or cell lines and may provide an important clue about potential tissue-specific biomarker candidates. To query our database, users can set their interested genomic region and choose the cut-off threshold to screen for the tissue-specific or ubiquitously expressed lncRNAs in lncExplore. Once the search parameters are submitted, the web page interface will return a list of lncRNAs that comply with the search criteria in descending order of tissue-specific score. Conversely, users can retrieve the ubiquitously expressed lncRNAs by entering the lower ‘Tau’ score. In the result pages, a summarized table for each lncRNA is presented with lncRNA ID, chromosome site and tissue-specific score. A hyperlink for each lncRNAs identifier is provided for users to cross-reference the basic sequence information and GO description. Also, a hyperlink in an expression column provides the expression signature for the corresponding transcripts with boxplots and bar plots. Therefore, users could examine whether the transcript shows the ‘spiked’ expression pattern among 24 cancers. The boxplot is available for download in various formats (PNG, JPEG and PDF).

#### Survival analysis

In the survival analysis section of lncExplore, based on gene expression signature, users can identify the potential biomarkers which are highly correlated with the survival time in a certain cancer type. lncExplore takes a quartile value for each transcript as a bifurcation point to conduct survival analysis for specific cancer datasets selected by users. By entering the transcript ID of interests and selecting quartile value and cancer type, the two groups (i.e. low-/high-expression groups) will be compared by Kaplan–Meier plots. When submitted, the Kaplan–Meier plot for user-selected cancer type will be displayed in different colors for two groups on the result page. Furthermore, we also list the *P*-value of log-rank test which was used to determine whether the survival curves among two groups are significantly different. Additionally, the hyperlink on each transcript ID in the summary table can lead the user to directly view the gene information and GO annotation. For example, the Kaplan–Meier plot of lncRNA *UCA1* (ncT0201182652) in colon adenocarcinoma shows the association of low- and high-expression levels (bifurcation point is median) with overall survival (Figure 3C). The *P*-value of log-rank test shows the results of comparing the survival distributions from two low- and high-expression groups for lncRNA *UCA1*. In lncRNA *UCA1*, the high expression group is associated with the lower survival rate than the low expression group. The survival information provides useful features for users to screen the potential biomarkers based on TCGA RNA-seq data.

#### Genomic physical interaction to identify the function of lncRNAs

Instead of predicting the lncRNA function by a co-expression network-based approach, we incorporated genome-wide characterization, such as *cis*-regulatory neighbors and miRNA-binding sites, to provide additional information for investigating uncharacterized lncRNAs. Thus, in lncExplore, we provide two major search categories for querying the lncRNAs of interest, including (i) eRNAs: lncRNAs produced from the enhancer region and potentially acting as eRNAs and (ii) ceRNAs: lncRNAs which act as post-transcription regulators for target competition within miRNA-regulated networks.

In the eRNAs section, we provide a list of genome-wide identified eRNAs based on the genomic information of the enhancer regions from VISTA database and Ensembl (43, 54). Therefore, by setting genomic regions in the search box of query pages, users can access eRNAs located in this specified region. The search result will be presented in a table which starts with lncRNA IDs, enhancer IDs, overlapping genomic coordinates and overlapping length. Since eRNAs regulate the transcription of neighbor genes and are critical to enhancer activity (27), we also included access to the information for neighbor genes by entering the range of genomic coordinate of interested IDs (Ensembl ID and ncT ID). The *cis*-regulatory element in the result page will show the potential regulator eRNAs for user interested genes.

In the ceRNA section, to help users understand the post-transcriptional regulatory networks of novel lncRNAs, we provide a list of precomputed interactions of miRNA and lncRNAs predicted by TargetScan (52). By entering either of the standard miRNA names (e.g. *hsa-miR-221-5p*) or lncRNAs ID in the search box, the user can easily browse the miRNA–lncRNA interaction relationships. A result table with the information for miRNA ID, lncRNAs ID and miRNA-binding sites will be presented. Users can further retrieve the detailed description of target lncRNAs, such as sequence and GO annotation, by clicking on our database ID (i.e. ‘ncT ID’ hyperlink). In addition, the result table for a search can be used for experimental validations to determine whether this lncRNA can serve as potential miRNA sponges or not.

## Discussion

Owing to the development of high-throughput sequencing and computational technology, many lncRNA databases have been developed for biologists to query and screen numerous disease-associated lncRNAs from RNA-seq data and literature, such as NONCODE v5, lncRNAdb, Lnc2Cancer and LncRNADisease (29–31, 33). However, most databases are literature-curated databases and focus on known lncRNAs. Table 1 lists the functional comparisons among our lncExplore and other published lncRNA databases. However, lncExplore has focused on observing expression profiles in known and novel lncRNAs across 24 human cancer datasets, and our database also integrates the survival analysis. Therefore, lncExplore has provided a useful platform for screening and finding potential pan-cancer lncRNA biomarkers.

**Table 1.** Comparison of existing lncRNA bioinformatics resources with lncExplore

**Name**	**Source of dataset**	**No. of disease**	**Description/Main features**	**Features of user interface**
lncExplore	TCGA, GEO	24	pan-cancer analysis across 24 diseases disease specificity and survival analysis for novel and known lncRNAs GO annotations for lncRNAs’ regulatory role information (eRNAs and ceRNAs)	user can screen novel lncRNAs by defining genomic location co-expression analysis could export searched results
lnCaNet	TCGA	11	pre-computed co-expression network between lncRNA and cancer-related RNAs lncRNA–cancer gene interaction 11 human cancers	−
starBase v2.0	CLIP-seq datasets	−	miRNA–RNA interactions by RNA-binding experiments functional annotation based on miRNA–target interaction	query by RNA–RNA interactions (i.e. miRNA–RNA and lncRNA–RNA)
TANRIC	TCGA	20	comprehensive clinical information for known lncRNAs analysis of user-defined RNAs somatic mutation information 20 human cancers	classify tumor subtypes by lncRNA expressions could export searched results
Lnc2Cancer	Literature	−	experimentally supported associations between lncRNAs and human cancers	classify regulatory mechanisms of lncRNAs in cancer
lncRNADisease 2.0	Literature	−	experimentally supported associations between lncRNAs and diseases	user can query by validated method
lncRNAdb v2	Human body map	−	manually curated reference database of eukaryotic lncRNAs	could export searched results BLAST search tool
NONCODE v5	Human body map	−	integrated annotation database of ncRNAs, especially lncRNAs lncRNA–disease associations based on literature evolutionary conservation information of lncRNAs	could export searched results Basic local alignment search tool (BLAST) search tool

In addition to collecting a greater number of cancer types and predicting novel lncRNAs, lncExplore provides advanced and distinctive features for lncRNA research in the following aspects. First, lncExplore is an integrative database that explores potential novel lncRNA–disease association by both pan-cancer and tissue-specific analysis based on RNA-seq data across 24 human cancers. Our pan-cancer analysis platform offers users more opportunities to find the common or aberrant lncRNA expression profiles across multiple cancer types. Second, lncExplore not only encompasses known lncRNAs, but also includes novel lncRNAs, which were predicted from TCGA RNA-seq data. In lncExplore, users can quickly access and screen genomic information about the predicted lncRNAs they are interested in from terabytes of RNA-seq data without understanding the use of the complex transcript prediction tools. Third, in general, the existing databases with respect to transcriptional regulation for lncRNAs, such as starBase and DIANA-LncBase, collected *in silico* analyses of lncRNA–miRNA interactions (35, 36). In contrast, lncExplore not only provides the predicted lncRNA–miRNA regulatory relationships, but also identifies the 7444 potential lncRNAs which are involved in enhancer-mediated gene expression and cancers. In addition, lncExplore provides a Pearson correlation coefficient tool that helps users explore extensive regulatory relationships between eRNAs and their neighboring genes (55). Fourth, lncExplore enables users to prioritize potential novel cancer-related lncRNAs or pan-cancer lncRNAs by integrating clinical survival analysis and disease specificity score. For novel potential lncRNA biomarkers, this clinical survival analysis improves the effectiveness from academic biological research to clinical practice. Fifth, lncExplore also provides users with GO annotations based on lncRNAs’ *cis*-regulatory elements. These associated terms may provide additional valuable insights into the molecular regulatory role of each lncRNA.

The major molecular characteristics of lncRNAs are non-coding transcripts and low sequence conservation between species (4). However, lncRNAs fulfill their regulatory functions via acting as competitive RNAs by competing for the shared miRNAs or as recruiters by binding to the transcriptional protein complex (2). Based on the *cis*-regulatory and ceRNA hypothesis, we predicted potential functional roles of novel and known lncRNAs in lncExplore by bioinformatics tools (GO enrichment analysis and miRNA target prediction) (52, 56). For example, *HOXA* was previously reported to be associated with many cancers, including breast cancer and colorectal cancer (57). According to GO enrichment results (BP and MF) from lncRNA *HOTTIP* neighbored genes, we found that the most statistically significantly enriched GO terms were associated with transcript regulatory, transcription factor activity, DNA binding, regionalization and embryonic morphogenesis (Supplementary Table S1). Those enriched terms are consistent with the well-established roles of lncRNA *HOTTIP* in *cis*-regulating the expression of *HOXA* (11). Interestingly, we also identified that the enriched BP GO terms of lncRNA *HOTTIP* neighbors were correlated with skeletal system morphogenesis. This feature will be the subject of further research. Therefore, our annotation pipeline in lncExplore actually provides clues to infer regulatory functions of lncRNAs, which are critically important to investigate the potential biological roles of novel lncRNAs.

As shown above, the lncExplore database integrates genomic profiles and clinical information to enable biologists to identify a set of cancer biomarker candidates at the molecular level. lncExplore provides a comprehensive resource for deciphering and analyzing cancer-related lncRNAs based on disease specificity score and predicted GO annotations and develops a user-friendly integrated platform for investigating the regulatory relationships of unannotated lncRNAs and potential associated genes, including protein-coding RNAs and miRNAs. Moreover, lncExplore was designed to be a powerful and intuitive web-based platform which supports a query from gene names (e.g. gene symbol and Ensembl ID) and genomic coordinates (support hg19 and hg38). While the biological data are growing faster than ever before, we believe that lncExplore will bridge the gap between biological and clinical information to help users gain valuable and global insights for improving real understanding of the complicated lncRNA regulatory mechanisms in human cancers and ultimately stimulate extended research for disease diagnosis and treatment.

## Materials and methods

### Data collection and preprocessing

#### Specialized databases for known lncRNAs

The lncExplore database hosts a comprehensive collection of lncRNAs including annotated sequences from public databases such as Ensembl (release 100), lncRNAdb, H-InvDB v9.0, NONCODE v5 and Human Body Map v2 (29, 41–43, 58, 59). Since Ensembl and NONCODE v5 are integrated databases that record various types of RNAs in different species, we extracted only human lncRNAs from these comprehensive databases.

#### High-throughput RNA-seq and Ribo-seq data

In the lncExplore database, there are 5034 RNA-seq datasets that were obtained from TCGA data portal (http://cancergenome.nih.gov/). All of these RNA-seq data have been preprocessed and aligned to the human genome (hg19). The cancer sample types of this RNA-seq data include primary solid tumor and metastases across 24 cancers. The corresponding patient clinical annotations for each cancer type were also retrieved by R package ‘TCGAbiolinks’ (60, 61). In addition, lncExplore also collected Ribo-seq data because ribosome-profiling techniques produce a global snapshot of all the actively translated genes in cells. The sequencing of these fragments indicates whether transcripts are being actively translated or not at a specific time point. The paired Ribo-seq and RNA-seq datasets for human samples were obtained from Andreev *et al.* and Gonzalez *et al.* studies for the evaluation of translation efficiency scores (62, 63). To obtain the high-quality reads from the raw data, the data were processed according to the following preprocessing procedures: trimming adapter sequencing and keeping reads that have at least 80% of bases with a quality score of more than 20. Moreover, these reads were aligned using bowtie v1.2 to the human genome (hg19) allowing for up to two mismatches, and unmapped reads were discarded (64).

#### Discovery of unannotated lncRNAs from high-throughput sequencing data

To discover novel lncRNAs from the RNA-seq data, all available RNA-seq data across 24 cancers were used to assemble transcripts by Cufflinks v2.2.0 with default parameters (65, 66). Subsequently, these transcripts were merged to give consensus sequences across cohorts by Cuffmerge. Moreover, to identify the unannotated novel transcripts from the merged transcriptome, Cuffcompare was used to compare these merged transcriptomes with the comprehensive gene references from five public databases: Ensembl, Human body map v2, NONCODE v5, H-InvDB v9.0 and lncRNAdb (29, 31, 42, 43, 58). Therefore, we focused on those transcripts which were classified into potentially novel transcripts and unknown intergenic transcripts by Cuffcompare. To ensure those unannotated novel transcripts are not protein coding, based on the definition of lncRNAs (1, 5), we discriminate potential unannotated lncRNAs from those novel transcripts through the following filtration processes: (i) selection of transcripts longer than 200 nucleotides; (ii) assessment of coding capacity of a putative non-coding transcript using CPAT v1.2 and txCds tools (67, 68); (iii) filtration of transcripts based on the translation efficiency score (69); (iv) filtration of the transcripts which are potential pseudogenes (Supplementary Figure S1). CPAT could estimate the coding potential of unannotated transcripts using alignment-free logistic regression model. According to CPAT results, for human transcripts, a score threshold of 0.364 gave the highest sensitivity and specificity. CPAT score < 0.364 indicates noncoding sequences. txCds is an ORF predictor from the University of California, Santa Cruz (UCSC) (68). Based on the recommendations of txCds, the transcripts with txCds score < 800 were considered non-coding transcripts. To get potential non-coding transcripts with the low-coding probability, we selected the transcripts which had both CPAT scores < 0.364 and txCds scores < 800. We adopted CPAT and txCds tools with default options for estimating human transcript coding probability. The translation efficiency of potential lncRNAs was calculated as the log2 ratio of Ribo-seq FPKM value to RNA-seq FPKM value (69). To extract the set of low translation efficiency non-coding transcripts, we used the threshold value (efficiency score ≤ upper quartile value) which was based on the distribution of translation efficiency score for GENCODE long non-coding transcripts. To remove potential pseudogenes from unannotated non-coding transcripts, we filtered transcripts which had 80% sequence identity by comparing them with GENCODE protein-coding gene sequences. Additionally, we provide hg19 and hg38 genomic coordinates for lncRNA query and demonstration. The genomic coordinates of novel lncRNAs were converted from hg19/GRCh37 to hg38/GRCh38 by using UCSC LiftOver tool (68). After filtering out most of the potential false-positive lncRNAs by using the multiple algorithms mentioned above, a set of highly confident lncRNAs predicted from different cancers were stored in the lncExplore database.

#### Survival analysis

The clinical information for 24 cancers in our database was obtained from TCGA project by R package ‘TCGAbiolinks’ (60, 61). To examine whether expression levels of lncRNAs were associated with cohort survival, the quantile value (q1, q2 or q3) of expression was considered as the cutoff value to divide lncRNAs into high and low expression groups in all cancers. Then, survival estimation and survival curves were calculated by Kaplan–Meier survival analysis based on the last follow-up time and the censor status from TCGA clinical datasets. Furthermore, we also reported the statistical results from log-rank test to compare the survival rate between lncRNA high- and low-expression groups. In our database, all RNA-seq data with available clinical information were used to estimate survival rate. The survival analysis was performed with R package ‘survival’ from Bioconductor (https://www.bioconductor.org/).

#### Predicting the function of lncRNAs by adjacent protein-coding gene

Due to the wide variety of functions and poor understanding of lncRNA-mediated process, it is generally difficult to infer their possible functions by the sequences compared with highly conserved protein-coding sequences. Nevertheless, previous studies have found that lncRNAs can regulate the expression of neighboring protein-coding genes (10, 70). Based on these studies, we searched the neighbor protein-coding genes which are within 100 kb upstream/downstream region of lncRNAs (45). We performed the GO enrichment analysis on the neighbor protein-coding gene set to predict the function of lncRNAs by R-package ‘topGO’ in our database (56). The *P*-value was adjusted by R tool p.adjust function (71). The lists of statistically significant GO terms (*P*-value < 0.05) of lncRNAs were stored in our database.

#### Identification of eRNAs

eRNAs are a class of lncRNAs and are transcribed from the enhancer regions (72). In our database, the loci of lncRNAs directly overlapped with known enhancer regions were considered potential eRNAs by using Bedtools (73). Then, for each eRNA, we reported its overlapped regions in lncExplore. The enhancer regions information was obtained from Ensembl and VISTA databases (https://enhancer.lbl.gov/) (43, 54).

#### Assessing disease specificity score

Previous studies have shown that lncRNAs possess tissue-specific expression features compared with protein-coding transcripts (3, 5). The tissue-specific expression features provide an important clue for screening potential tissue-specific biomarkers from a comprehensive collection of 24 human cancer datasets. Therefore, in lncExplore, we calculated a tissue-specific score for each lncRNA based on the ‘Tau’ score using TCGA RNA-seq data (44). The ‘Tau’ score ranging from 0 to 1 represents a degree to which an lncRNA is expressionally specific to a particular tissue. This disease specificity score (‘tau’ value) was calculated as follows:

\begin{equation*}{\rm{\tau }} = {{\mathop \sum \nolimits_{i = 1}^n \left( {1 - \hat x} \right)} \over {n - 1}};\hat x = {{{x_i}} \over {\mathop {\max }\limits_{i \le i \le n} \left( {{x_i}} \right)}};\end{equation*}

where *n* represents the number of diseases/tissues and $ \hat x$ represents the FPKM value of the transcripts in the *i*-th diseases/tissues normalized by the maximal expression value. For example, values close to 1 show transcripts expressed specifically in only one disease/tissue and values close to 0 show transcripts expressed ubiquitously in many diseases/tissues.

#### Pairwise RNA-seq dataset

The sample size of RNA-seq datasets for pairwise comparisons across six diseases in lncExplore: (i) breast invasive carcinoma: 106, (ii) colon adenocarcinoma: 34, (iii) kidney renal clear cell carcinoma: 138, (iv) lung adenocarcinoma: 102, (v) lung squamous cell carcinoma: 88 and (vi) prostate adenocarcinoma: 98. The gene expressions for pairwise datasets were analyzed by StringTie (74).

## Supplementary Material

baab053_Supp

## References

[1] Fu X.D. (2014) Non-coding RNA: a new frontier in regulatory biology. *Natl. Sci. Rev.*, 1, 190–204. 25821635 10.1093/nsr/nwu008 PMC4374487

[2] Marchese F.P. , Raimondi I. and Huarte M. (2017) The multidimensional mechanisms of long noncoding RNA function. *Genome Biol.*, 18, 206. 10.1186/s13059-017-1348-2 PMC5663108 29084573

[3] Ward M. , McEwan C., Mills J.D. et al. (2015) Conservation and tissue-specific transcription patterns of long noncoding RNAs. *J. Hum. Transcriptome*, 1, 2–9. 10.3109/23324015.2015.1077591 PMC4894084 27335896

[4] Johnsson P. , Lipovich L., Grander D. et al. (2014) Evolutionary conservation of long non-coding RNAs; sequence, structure, function. *Biochim. Biophys. Acta*, 1840, 1063–1071. 24184936 10.1016/j.bbagen.2013.10.035 PMC3909678

[5] Derrien T. , Johnson R., Bussotti G. et al. (2012) The GENCODE v7 catalog of human long noncoding RNAs: analysis of their gene structure, evolution, and expression. *Genome Res.*, 22, 1775–1789. 22955988 10.1101/gr.132159.111 PMC3431493

[6] Wang K.C. and Chang H.Y. (2011) Molecular mechanisms of long noncoding RNAs. *Mol. Cell*, 43, 904–914. 21925379 10.1016/j.molcel.2011.08.018 PMC3199020

[7] St Laurent G. , Wahlestedt C. and Kapranov P. (2015) The landscape of long noncoding RNA classification. *Trends Genet.*, 31, 239–251. 25869999 10.1016/j.tig.2015.03.007 PMC4417002

[8] Yoon J.H. , Abdelmohsen K. and Gorospe M. (2013) Posttranscriptional gene regulation by long noncoding RNA. *J. Mol. Biol.*, 425, 3723–3730. 23178169 10.1016/j.jmb.2012.11.024 PMC3594629

[9] Guil S. and Esteller M. (2012) Cis-acting noncoding RNAs: friends and foes. *Nat. Struct. Mol. Biol.*, 19, 1068–1075. 23132386 10.1038/nsmb.2428

[10] Joung J. , Engreitz J.M., Konermann S. et al. (2017) Genome-scale activation screen identifies a lncRNA locus regulating a gene neighbourhood. *Nature*, 548, 343–346. 28792927 10.1038/nature23451 PMC5706657

[11] Wang K.C. , Yang Y.W., Liu B. et al. (2011) A long noncoding RNA maintains active chromatin to coordinate homeotic gene expression. *Nature*, 472, 120–124. 21423168 10.1038/nature09819 PMC3670758

[12] Guttman M. and Rinn J.L. (2012) Modular regulatory principles of large non-coding RNAs. *Nature*, 482, 339–346. 22337053 10.1038/nature10887 PMC4197003

[13] Froberg J.E. , Yang L. and Lee J.T. (2013) Guided by RNAs: X-inactivation as a model for lncRNA function. *J. Mol. Biol.*, 425, 3698–3706. 23816838 10.1016/j.jmb.2013.06.031 PMC3771680

[14] Fatica A. and Bozzoni I. (2014) Long non-coding RNAs: new players in cell differentiation and development. *Nat. Rev. Genet.*, 15, 7–21. 24296535 10.1038/nrg3606

[15] Romero-Barrios N. , Legascue M.F., Benhamed M. et al. (2018) Splicing regulation by long noncoding RNAs. *Nucleic Acids Res.*, 46, 2169–2184. 29425321 10.1093/nar/gky095 PMC5861421

[16] Zhao Y. , Sun H. and Wang H. (2016) Long noncoding RNAs in DNA methylation: new players stepping into the old game. *Cell Biosci.*, 6, 45. 10.1186/s13578-016-0109-3 PMC4940868 27408682

[17] Yoon J.H. , Abdelmohsen K., Kim J. et al. (2013) Scaffold function of long non-coding RNA HOTAIR in protein ubiquitination. *Nat. Commun.*, 4, 2939. 10.1038/ncomms3939 PMC4556280 24326307

[18] Balas M.M. and Johnson A.M. (2018) Exploring the mechanisms behind long noncoding RNAs and cancer. *Non-Coding RNA Res.*, 3, 108–117. 10.1016/j.ncrna.2018.03.001 PMC6114262 30175284

[19] Spizzo R. , Almeida M., Colombatti A. et al. (2012) Long non-coding RNAs and cancer: a new frontier of translational research&quest. *Oncogene*, 31, 4577–4587. 22266873 10.1038/onc.2011.621 PMC3433647

[20] Li H. , Yu B., Li J. et al. (2014) Overexpression of lncRNA H19 enhances carcinogenesis and metastasis of gastric cancer. *Oncotarget*, 5, 2318–2329. 24810858 10.18632/oncotarget.1913 PMC4039165

[21] Chan J.J. and Tay Y. (2018) Noncoding RNA:RNA regulatory networks in cancer. *Int. J. Mol. Sci.*, 19, 1310. 10.3390/ijms19051310 PMC5983611 29702599

[22] Hayes E.L. and Lewis-Wambi J.S. (2015) Mechanisms of endocrine resistance in breast cancer: an overview of the proposed roles of noncoding RNA. *Breast Cancer Res. BCR*, 17, 40. 10.1186/s13058-015-0542-y PMC4362832 25849966

[23] Godinho M.F. , Sieuwerts A.M., Look M.P. et al. (2010) Relevance of BCAR4 in tamoxifen resistance and tumour aggressiveness of human breast cancer. *Br. J. Cancer*, 103, 1284–1291. 20859285 10.1038/sj.bjc.6605884 PMC2967058

[24] Collette J. , Le Bourhis X. and Adriaenssens E. (2017) Regulation of human breast cancer by the long non-coding RNA H19. *Int. J. Mol. Sci.*, 18, 2319. 10.3390/ijms18112319 PMC5713288 29099749

[25] Luo M. , Li Z., Wang W. et al. (2013) Long non-coding RNA H19 increases bladder cancer metastasis by associating with EZH2 and inhibiting E-cadherin expression. *Cancer Lett.*, 333, 213–221. 23354591 10.1016/j.canlet.2013.01.033

[26] Lam M.T. , Li W., Rosenfeld M.G. et al. (2014) Enhancer RNAs and regulated transcriptional programs. *Trends Biochem. Sci.*, 39, 170–182. 24674738 10.1016/j.tibs.2014.02.007 PMC4266492

[27] Li W. , Notani D. and Rosenfeld M.G. (2016) Enhancers as non-coding RNA transcription units: recent insights and future perspectives. *Nat. Rev. Genet.*, 17, 207–223. 26948815 10.1038/nrg.2016.4

[28] Dinger M.E. , Pang K.C., Mercer T.R. et al. (2009) NRED: a database of long noncoding RNA expression. *Nucleic Acids Res.*, 37, D122–126. 18829717 10.1093/nar/gkn617 PMC2686506

[29] Fang S. , Zhang L., Guo J. et al. (2018) NONCODEV5: a comprehensive annotation database for long non-coding RNAs. *Nucleic Acids Res.*, 46, D308–D314. 29140524 10.1093/nar/gkx1107 PMC5753287

[30] Gao Y. , Wang P., Wang Y. et al. (2019) Lnc2Cancer v2.0: updated database of experimentally supported long non-coding RNAs in human cancers. *Nucleic Acids Res.*, 47, D1028–D1033. 30407549 10.1093/nar/gky1096 PMC6324001

[31] Quek X.C. , Thomson D.W., Maag J.L. et al. (2015) lncRNAdb v2.0: expanding the reference database for functional long noncoding RNAs. *Nucleic Acids Res.*, 43, D168–173. 25332394 10.1093/nar/gku988 PMC4384040

[32] Park C. , Yu N., Choi I. et al. (2014) lncRNAtor: a comprehensive resource for functional investigation of long non-coding RNAs. *Bioinformatics*, 30, 2480–2485. 24813212 10.1093/bioinformatics/btu325

[33] Chen G. , Wang Z., Wang D. et al. (2013) LncRNADisease: a database for long-non-coding RNA-associated diseases. *Nucleic Acids Res.*, 41, D983–986. 23175614 10.1093/nar/gks1099 PMC3531173

[34] Zhou B. , Zhao H., Yu J. et al. (2018) EVLncRNAs: a manually curated database for long non-coding RNAs validated by low-throughput experiments. *Nucleic Acids Res.*, 46, D100–D105. 28985416 10.1093/nar/gkx677 PMC5753334

[35] Paraskevopoulou M.D. , Georgakilas G., Kostoulas N. et al. (2013) DIANA-LncBase: experimentally verified and computationally predicted microRNA targets on long non-coding RNAs. *Nucleic Acids Res.*, 41, D239–245. 23193281 10.1093/nar/gks1246 PMC3531175

[36] Li J.H. , Liu S., Zhou H. et al. (2013) starBase v2.0: decoding miRNA-ceRNA, miRNA-ncRNA and protein-RNA interaction networks from large-scale CLIP-Seq data. *Nucleic Acids Res.*, 42, D92–97. 24297251 10.1093/nar/gkt1248 PMC3964941

[37] Lee H. , Palm J., Grimes S.M. et al. (2015) The cancer genome atlas clinical explorer: a web and mobile interface for identifying clinical-genomic driver associations. *Genome Med.*, 7, 112. 10.1186/s13073-015-0226-3 PMC4624593 26507825

[38] Shi T. , Gao G. and Cao Y. (2016) Long noncoding RNAs as novel biomarkers have a promising future in cancer diagnostics. *Dis. Markers*, 2016, 9085195. 10.1155/2016/9085195 PMC4842029 27143813

[39] Andersson R. , Gebhard C., Miguel-Escalada I. et al. (2014) An atlas of active enhancers across human cell types and tissues. *Nature*, 507, 455–461. 24670763 10.1038/nature12787 PMC5215096

[40] Sanyal A. , Lajoie B.R., Jain G. et al. (2012) The long-range interaction landscape of gene promoters. *Nature*, 489, 109–113. 22955621 10.1038/nature11279 PMC3555147

[41] Amaral P.P. , Clark M.B., Gascoigne D.K. et al. (2011) lncRNAdb: a reference database for long noncoding RNAs. *Nucleic Acids Res.*, 39, D146–D151. 21112873 10.1093/nar/gkq1138 PMC3013714

[42] Yamasaki C. , Murakami K., Fujii Y. et al. (2008) The H-Invitational Database (H-InvDB), a comprehensive annotation resource for human genes and transcripts. *Nucleic Acids Res.*, 36, D793–799. 18089548 10.1093/nar/gkm999 PMC2238988

[43] Cunningham F. , Achuthan P., Akanni W. et al. (2019) Ensembl 2019. *Nucleic Acids Res.*, 47, D745–D751. 30407521 10.1093/nar/gky1113 PMC6323964

[44] Yanai I. , Benjamin H., Shmoish M. et al. (2005) Genome-wide midrange transcription profiles reveal expression level relationships in human tissue specification. *Bioinformatics*, 21, 650–659. 15388519 10.1093/bioinformatics/bti042

[45] Zhu Y. , Mao D., Gao W. et al. (2019) Analysis of lncRNA expression in patients with eosinophilic and neutrophilic asthma focusing on LNC_000127. *Front. Genet.*, 10, 141. 10.3389/fgene.2019.00141 PMC6433975 30941157

[46] Quagliata L. , Matter M.S., Piscuoglio S. et al. (2014) Long noncoding RNA HOTTIP/HOXA13 expression is associated with disease progression and predicts outcome in hepatocellular carcinoma patients. *Hepatology*, 59, 911–923. 24114970 10.1002/hep.26740 PMC3943759

[47] Li Z. , Zhao X., Zhou Y. et al. (2015) The long non-coding RNA HOTTIP promotes progression and gemcitabine resistance by regulating HOXA13 in pancreatic cancer. *J. Transl. Med.*, 13, 84. 10.1186/s12967-015-0442-z PMC4372045 25889214

[48] Lian Y. , Cai Z., Gong H. et al. (2016) HOTTIP: a critical oncogenic long non-coding RNA in human cancers. *Mol. Biosyst.*, 12, 3247–3253. 27546609 10.1039/c6mb00475j

[49] Espinosa J.M. (2017) On the origin of lncRNAs: missing link found. *Trends Genet.*, 33, 660–662. 28778681 10.1016/j.tig.2017.07.005 PMC5610073

[50] Hirano T. , Yoshikawa R., Harada H. et al. (2015) Long noncoding RNA, CCDC26, controls myeloid leukemia cell growth through regulation of KIT expression. *Mol. Cancer*, 14, 90. 10.1186/s12943-015-0364-7 PMC4423487 25928165

[51] Chen C. , Wang P., Mo W. et al. (2019) lncRNA-CCDC26, as a novel biomarker, predicts prognosis in acute myeloid leukemia. *Oncol. Lett.*, 18, 2203–2211. 31452721 10.3892/ol.2019.10591 PMC6676650

[52] Agarwal V. , Bell G.W., Nam J.W. et al. (2015) Predicting effective microRNA target sites in mammalian mRNAs. *eLife*, 4, e05005. 10.7554/eLife.05005 PMC4532895 26267216

[53] Gong P. , Qiao F., Wu H. et al. (2018) LncRNA UCA1 promotes tumor metastasis by inducing miR-203/ZEB2 axis in gastric cancer. *Cell Death Dis.*, 9, 1158. 10.1038/s41419-018-1170-0 PMC6249325 30464170

[54] Visel A. , Minovitsky S., Dubchak I. et al. (2007) VISTA enhancer browser—a database of tissue-specific human enhancers. *Nucleic Acids Res.*, 35, D88–92. 17130149 10.1093/nar/gkl822 PMC1716724

[55] Qi W. , Song X. and Li L. (2013) Long non-coding RNA-guided regulation in organisms. *Sci. China Life Sci.*, 56, 891–896. 24091686 10.1007/s11427-013-4558-1

[56] Alexa A. and Rahnenfuhrer J. (2010) topGO: topGO: enrichment analysis for gene ontology.

[57] Bhatlekar S. , Fields J.Z. and Boman B.M. (2018) Role of HOX Genes in stem cell differentiation and cancer. *Stem Cells Int*, 2018, 3569493. 10.1155/2018/3569493 PMC6081605 30154863

[58] Zhao Y. , Li H., Fang S. et al. (2015) NONCODE 2016: an informative and valuable data source of long non-coding RNAs. *Nucleic Acids Res.*, 44, D203–208. 26586799 10.1093/nar/gkv1252 PMC4702886

[59] Kersey P.J. , Allen J.E., Armean I. et al. (2016) Ensembl genomes 2016: more genomes, more complexity. *Nucleic Acids Res.*, 44, D574–580. 26578574 10.1093/nar/gkv1209 PMC4702859

[60] Colaprico A. , Silva T.C., Olsen C. et al. (2016) TCGAbiolinks: an R/Bioconductor package for integrative analysis of TCGA data. *Nucleic Acids Res.*, 44, e71. 10.1093/nar/gkv1507 PMC4856967 26704973

[61] Mounir M. , Lucchetta M., Silva T.C. et al. (2019) New functionalities in the TCGAbiolinks package for the study and integration of cancer data from GDC and GTEx. *PLoS Comput. Biol.*, 15, e1006701. 10.1371/journal.pcbi.1006701 PMC6420023 30835723

[62] Andreev D.E. , O’Connor P.B., Fahey C. et al. (2015) Translation of 5’ leaders is pervasive in genes resistant to eIF2 repression. *eLife*, 4, e03971. 10.7554/eLife.03971 PMC4383229 25621764

[63] Gonzalez C. , Sims J.S., Hornstein N. et al. (2014) Ribosome profiling reveals a cell-type-specific translational landscape in brain tumors. *J. Neurosci.*, 34, 10924–10936. 25122893 10.1523/JNEUROSCI.0084-14.2014 PMC4131009

[64] Langmead B. , Trapnell C., Pop M. et al. (2009) Ultrafast and memory-efficient alignment of short DNA sequences to the human genome. *Genome Biol.*, 10, R25. 10.1186/gb-2009-10-3-r25 PMC2690996 19261174

[65] Trapnell C. , Williams B.A., Pertea G. et al. (2010) Transcript assembly and quantification by RNA-Seq reveals unannotated transcripts and isoform switching during cell differentiation. *Nat. Biotechnol.*, 28, 511–515. 20436464 10.1038/nbt.1621 PMC3146043

[66] Trapnell C. , Roberts A., Goff L. et al. (2012) Differential gene and transcript expression analysis of RNA-seq experiments with TopHat and Cufflinks. *Nat. Protoc.*, 7, 562–578. 22383036 10.1038/nprot.2012.016 PMC3334321

[67] Wang L. , Park H.J., Dasari S. et al. (2013) CPAT: coding-potential assessment tool using an alignment-free logistic regression model. *Nucleic Acids Res.*, 41, e74. 10.1093/nar/gkt006 PMC3616698 23335781

[68] Haeussler M. , Zweig A.S., Tyner C. et al. (2019) The UCSC genome browser database: 2019 update. *Nucleic Acids Res.*, 47, D853–D858. 30407534 10.1093/nar/gky1095 PMC6323953

[69] Ji Z. , Song R., Regev A. et al. (2015) Many lncRNAs, 5ʹUTRs, and pseudogenes are translated and some are likely to express functional proteins. *eLife*, 4, e08890. 10.7554/eLife.08890 PMC4739776 26687005

[70] Goff L.A. , Groff A.F., Sauvageau M. et al. (2015) Spatiotemporal expression and transcriptional perturbations by long noncoding RNAs in the mouse brain. *Proc. Natl. Acad. Sci. U.S.A.*, 112, 6855–6862. 26034286 10.1073/pnas.1411263112 PMC4460505

[71] Benjamini Y. and Hochberg Y. (1995) Controlling the false discovery rate: a practical and powerful approach to multiple testing. *J. R. Stat. Soc. Series B*, 57, 289–300.

[72] Schaukowitch K. , Joo J.-Y., Liu X. et al. (2014) Enhancer RNA facilitates NELF release from immediate early genes. *Mol. Cell*, 56, 29–42. 25263592 10.1016/j.molcel.2014.08.023 PMC4186258

[73] Quinlan A.R. (2014) BEDTools: the Swiss-army tool for genome feature analysis. *Curr. Protoc. Bioinf.*, 47, 11.12.1–11.12.34. 10.1002/0471250953.bi1112s47 PMC4213956 25199790

[74] Pertea M. , Kim D., Pertea G.M. et al. (2016) Transcript-level expression analysis of RNA-seq experiments with HISAT, StringTie and Ballgown. *Nat. Protoc.*, 11, 1650–1667. 27560171 10.1038/nprot.2016.095 PMC5032908

