# Supplementary material for: lncExplore: a database of pan-cancer analysis and systematic functional annotation for lncRNAs from RNA-sequencing data
Source: Database (Oxford). 2021 Aug 31;2021:baab053. doi: 10.1093/database/baab053 (PMC8407485; doi:10.1093/database/baab053)
Supplement: baab053_Supp [file baab053_supp.zip › Supp. Figure 1.pptx]

## Slide 1
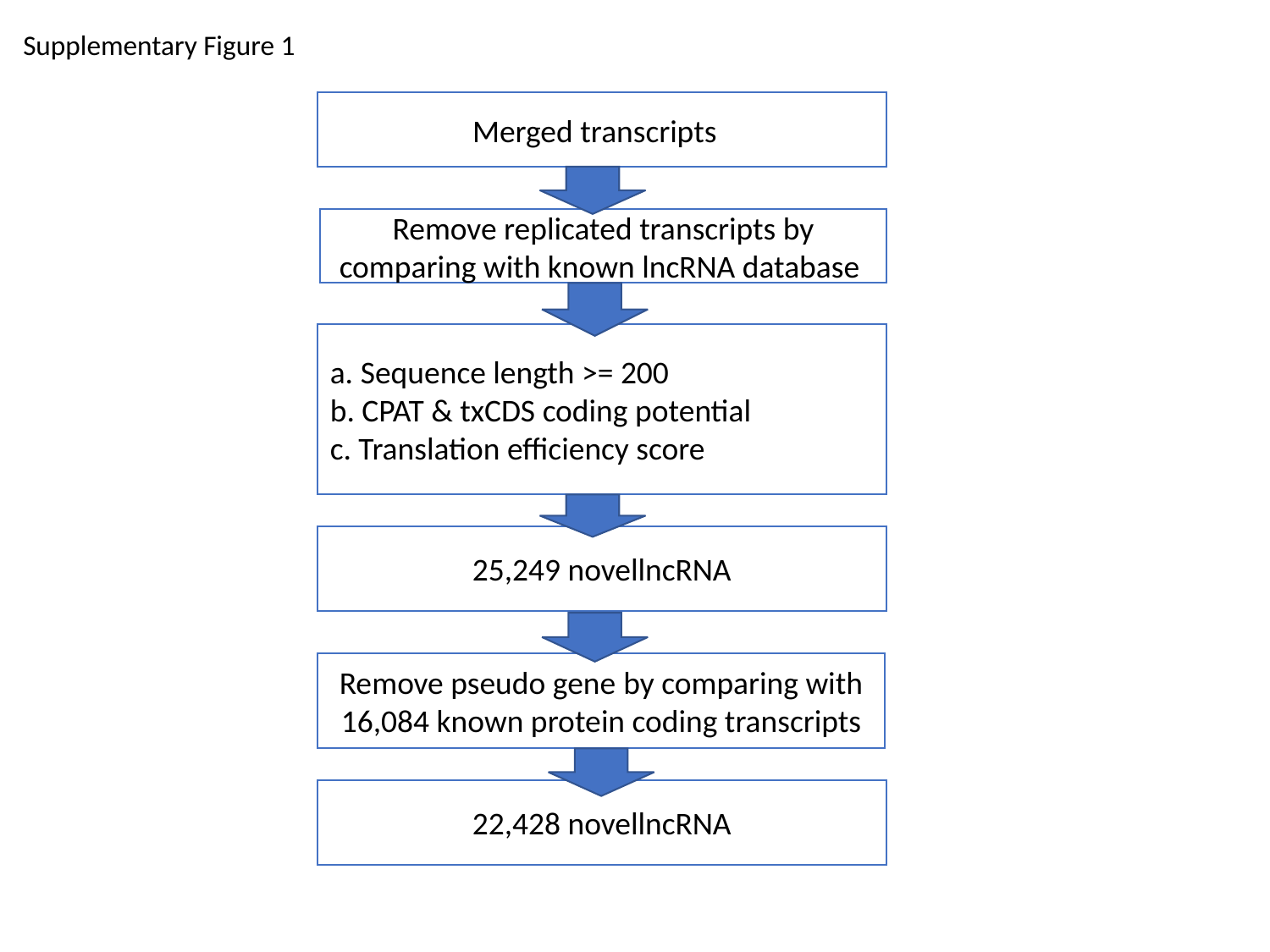

Supplementary Figure 1
Merged transcripts
Remove replicated transcripts by comparing with known lncRNA database
a. Sequence length >= 200
b. CPAT & txCDS coding potential
c. Translation efficiency score
25,249 novellncRNA
Remove pseudo gene by comparing with 16,084 known protein coding transcripts
22,428 novellncRNA
