# Supplementary material for: lncExplore: a database of pan-cancer analysis and systematic functional annotation for lncRNAs from RNA-sequencing data
Source: Database (Oxford). 2021 Aug 31;2021:baab053. doi: 10.1093/database/baab053 (PMC8407485; doi:10.1093/database/baab053)
Supplement: baab053_Supp [file baab053_supp.zip › Table_S1.docx]

Supplementary Table1: Top10 statistically significant Gene Ontology terms from enriched analysis of lncRNAs *HOTTIP* neighbored genes

| **GO-term** | **Description** | ***P*-value** | ***q-value*** |
| --- | --- | --- | --- |
| **Molecular Function** |  |  |  |
| GO:0000981 | RNA polymerase II transcription factor activity, sequence-specific DNA binding | 7.70E-12 | 0 |
| GO:0003700 | transcription factor activity, sequence-specific DNA binding | 1.30E-11 | 0 |
| GO:0140110 | transcription regulator activity | 8.60E-11 | 0 |
| GO:0043565 | sequence-specific DNA binding | 4.60E-07 | 0.00034 |
| GO:0003677 | DNA binding | 0.00014 | 0.07216 |
| GO:0000978 | RNA polymerase II core promoter proximal region sequence-specific DNA binding | 0.00015 | 0.07216 |
| GO:0000987 | core promoter proximal region sequence-specific DNA binding | 0.00017 | 0.07216 |
| GO:0000977 | RNA polymerase II regulatory region sequence-specific DNA binding | 0.0005 | 0.16517 |
| GO:0001012 | RNA polymerase II regulatory region DNA binding | 0.0005 | 0.16517 |
| GO:0000976 | transcription regulatory region sequence-specific DNA binding | 0.00066 | 0.19406 |
|  |  |  |  |
|  |  |  |  |
| **Biological Process** |  |  |  |
| GO:0001501 | skeletal system development | 8.10E-13 | 0 |
| GO:0048706 | embryonic skeletal system development | 5.20E-11 | 0 |
| GO:0048704 | embryonic skeletal system morphogenesis | 1.50E-09 | 1.00E-05 |
| GO:0048598 | embryonic morphogenesis | 1.70E-08 | 5.00E-05 |
| GO:0009790 | embryo development | 2.20E-08 | 5.00E-05 |
| GO:0003002 | regionalization | 2.40E-08 | 5.00E-05 |
| GO:0009792 | embryo development ending in birth or egg hatching | 2.70E-08 | 5.00E-05 |
| GO:0007389 | pattern specification process | 1.10E-07 | 0.00017 |
| GO:0048705 | skeletal system morphogenesis | 1.30E-07 | 0.00018 |
| GO:0060065 | uterus development | 3.20E-07 | 0.00041 |
|  |  |  |  |
